# Gonorrhœa Again

**Published:** 1891-04

**Authors:** I. Dever

**Affiliations:** Clinton, N. Y.


### GONORRHŒA AGAIN.

EDITORS HOMŒOPATHIC PHYSICIAN:

The February number of your journal contains an article from the pen of Dr. White, in which he expresses dissappointment with my paper on gonorrhœa and syphilis. He failed to give us the advantage of his criticism, but dispensed with chronic gonorrhœa in a way which would lead those who are not experienced in the treatment of those troublesome difficulties to think that a chronic gonorrhœa kept up by some constitutional dyscrasia is more amenable to treatment than an acute case of the same difficulty.

Granting this to be true, no better apology need be offered for the harsh and unnatural treatment instituted by eclectic homœopaths, who, by injections of caustic medicines, have but little difficulty in converting their acute cases of gonorrhœa into the chronic form.

It is a matter of doubt with some able physicians whether an individual in whom there is no constitutional taint will contract gonorrhœa, though many times exposed to the influence of the poison. I am not satisfied in regard to the truth of the above proposition, but I do know that an otherwise healthy subject can be readily cured of acute gonorrhœa by following Hahnemann's directions, which will also answer the Doctor's question, " How are we to treat or to cure a typical case of acute gonorrhœa?"

Hahnemann has told us in his *Organon* that " The totality of the symptoms constitutes the only indication for the selection of the remedy." A remedy selected in strict obedience to the above directions will not only cure the acute gonorrhœa, but the constitutional dyscrasia will also disappear, and the patient will often tell his physician, " I am in better health than I was before I had the gonorrhœa."

I will admit that all cases do not make a rapid recovery, and that some are tedious in the hands of the best prescribers; nevertheless this is no excuse for those who abandon a law of cure for the *rule of cut and try*, and then attempt to explain away every cure to which they cannot apply the rule.

"Are not injections of a homœopathic solution of Mercury equivalent to the dose by the mouth, and has it any preference to the Mercury given by the mouth?" This is a question worthy of the attention of those who are in the habit of using injections of Mercury in gonorrhœa; but before they reach a final conclusion I would advise them to study the physiological uses of both the mouth and the urethra, which present marked differences in their relation to the animal economy.

Mercury, like all other remedies, can only be " homœopathic Mercury " when indicated by the totality of the symptoms—

subjective and objective—otherwise it would not be the homœopathic remedy, though prescribed in the CM potency. I would not use injections of a solution of "homœopathic Mercury" in a case of gonorrhœa for the same reason that I would not prescribe a local application of Arsenicum for the cure of a case of crusta lactea. I would be prescribing one remedy for the cure of a disease in which some other remedy might be indicated—worse than that—I would be throwing an obstruction in the way of Nature's cure, which Hering tells us proceeds from the centre to the circumference—from above downward. I admire Dr. White's honest confession; and, as his paper was undoubtedly written as a draw, I have taken the liberty to answer with this paper.

I. Dever.

Clinton, N. Y., March 4th, 1891.

